# Intrinsic Properties of Brown and White Adipocytes Have Differential Effects on Macrophage Inflammatory Responses

**DOI:** 10.1155/2017/9067049

**Published:** 2017-03-26

**Authors:** Louisa Dowal, Pooja Parameswaran, Sarah Phat, Syamala Akella, Ishita Deb Majumdar, Jyoti Ranjan, Chahan Shah, Saie Mogre, Kalyani Guntur, Khampaseuth Thapa, Stephane Gesta, Vivek K. Vishnudas, Niven R. Narain, Rangaprasad Sarangarajan

**Affiliations:** Berg LLC, Framingham, MA, USA

## Abstract

Obesity is marked by chronic, low-grade inflammation. Here, we examined whether intrinsic differences between white and brown adipocytes influence the inflammatory status of macrophages. White and brown adipocytes were characterized by transcriptional regulation of *UCP-1*, *PGC1α*, *PGC1β*, and *CIDEA* and their level of IL-6 secretion. The inflammatory profile of PMA-differentiated U937 and THP-1 macrophages, in resting state and after stimulation with LPS/IFN-gamma and IL-4, was assessed by measuring IL-6 secretion and transcriptional regulation of a panel of inflammatory genes after mono- or indirect coculture with white and brown adipocytes. White adipocyte monocultures show increased IL-6 secretion compared to brown adipocytes. White adipocytes cocultured with U937 and THP-1 macrophages induced a greater increase in IL-6 secretion compared to brown adipocytes cocultured with both macrophages. White adipocytes cocultured with macrophages increased inflammatory gene expression in both types. In contrast, macrophages cocultured with brown adipocytes induced downregulation or no alterations in inflammatory gene expression. The effects of adipocytes on macrophages appear to be independent of stimulation state. Brown adipocytes exhibit an intrinsic ability to dampen inflammatory profile of macrophages, while white adipocytes enhance it. These data suggest that brown adipocytes may be less prone to adipose tissue inflammation that is associated with obesity.

## 1. Introduction

Obesity is a major health burden affecting nearly 78.6 million Americans [1]. It is characterized by pathophysiological changes, including hypertrophy and hyperplasia of adipocytes and chronic, low-grade systemic inflammation [2, 3]. Of particular interest is the contribution of low-grade inflammation to insulin resistance, metabolic disorders, and cardiovascular complications associated with obesity [4, 5].

Obesity is marked by increased levels of circulating inflammatory factors and increased infiltration of macrophages into adipose tissue, in particular white adipose tissue (WAT) [2, 6]. Adipose tissue is predominantly comprised of adipocytes but also contains various cell types, which includes preadipocytes, fibroblasts, vascular endothelial cells, and a variety of immune cells, such as macrophages. Traditionally, adipose tissue (AT) has been classified as WAT and brown adipose tissue (BAT) and is characteristically defined based on its distinct physiological functions. Specifically, WAT functions to store energy in the form of lipids, while BAT is involved in energy expenditure via thermogenesis [7]. However, recent work has indicated that WAT can undergo a process called browning [8], in which an inducible brown adipocyte (also called beige, brown-in-white, or brite adipocyte) develops [9]. While beige adipocytes have been described to be phenotypically distinct from classic brown and white adipocytes [9], in terms of functionality, that is, regulation of metabolism, beige adipocytes are similar to brown adipocytes (10).

WAT is distributed in visceral and thoracic cavities and subcutaneously in the abdomen and extremities, while BAT has been described to be present and plays a significant physiological role in fetuses and neonates [10–12]. Until recently, BAT was thought to have limited physiological function in adults, but imaging studies have revealed metabolically active BAT in interscapular and cervical regions of normal adults [13, 14]. This is consistent with the high levels of mitochondria in BAT, which uniquely express high levels of the inner mitochondrial membrane protein, uncoupling protein 1 (UCP-1), also known as thermogenin [15]. In mouse models of diet-induced obesity (DIO), macrophage infiltration and inflammation in BAT have been observed [16]. In adipose tissue of DIO mice, activation of classically activated macrophages has been shown to suppress the induction of UCP-1. Of interest is that macrophage infiltration in BAT was found to be significantly less than that in WAT [16] and enhanced mRNA expression of inflammatory molecules, including tumor necrosis factor (TNF) *α*, interleukin-6 (IL-6), and monocyte chemotactic protein (MCP-1), was nearly 10–100-fold less in BAT than that in WAT in DIO mice. In addition, others have shown that expression of proinflammatory cytokines is lower in BAT than in WAT in normal mice [17] and expression of several cytokines has been found to be lower in C2D macrophage cells that were isolated after in vivo injection into BAT compared to injection into WAT [17]. However, as adipose tissue is a heterogeneous organ, it is unknown which specific type(s) of cell(s) in the adipose tissue environment is(are) contributing to the observed differences between WAT and BAT.

The present study attempted to address whether differential inflammatory cues that originate from differentiated brown and white adipocytes could be observed in isolated conditions outside the tissue environment. Specifically, in these studies, adipocytes were cocultured with differentiated U937 or THP-1 cells, two monocytic cell lines that represent well established, in vitro macrophage models. Using this approach, we revealed that coculture of macrophages with white adipocytes increases IL-6 secretion and induces expression of a set of genes in macrophages, known to be involved in regulation of inflammation. However, coculture of macrophages with brown adipocytes resulted in a minimal and nonsignificant release in IL-6 secretion and a general reduction or lack of induction in expression of the genes regulating inflammation in macrophages. Together, these data demonstrate that brown adipocytes exhibit intrinsic properties which make them less prone to the promotion of an inflammatory environment for macrophages, when compared to white adipocytes. Thus, therapeutic strategies aiming to increase levels of BAT to protect against obesity may be beneficial by not only promoting energy expenditure [18, 19] but also reducing inflammatory burden.

## 2. Materials and Methods

### 2.1. Monocyte Cell Culture and Differentiation

U937 monocytes (Sigma) were cultured in Roswell Park Memorial Institute medium (RPMI) 1640 supplemented with 10% fetal bovine serum (FBS) and penicillin/streptomycin (Pen/Strep), and THP-1 monocytes (Sigma) were cultured in RPMI-1640 supplemented with 10% FBS, 1 mM sodium pyruvate, and Pen/Strep. Both cell lines were maintained at a density of 5–8 × 10^5^ cells/ml and were subcultured every 3-4 days to maintain cells in the logarithmic phase of growth.

Monocytes were seeded in 6-well plates (9.5 cm^2^) for differentiation at 500,000 cells/well for U937 and 750,000 cells/well for THP-1. This corresponds to 53,000 cells/cm^2^ for U937 and 79,000 cells/cm^2^ for THP-1. For differentiation, monocytes were cultured in 20 nM phorbol 12-myristate 13-acetate (PMA) for 72 hours, and then media was exchanged to PMA-free culture media. The macrophages were left untreated (i.e., allowed to rest) or were stimulated with pro- or anti-inflammatory stimuli (see below for details) for 24 hours before use in cellular assays. Differentiation into macrophages was confirmed by flow cytometric analysis of the cell surface markers CD11b (BD Biosciences) and CD11c (BD Biosciences) and by an increase in phagocytic activity of pHrodo Red Zymosan BioParticles Conjugates (Life Technologies) (see Supplemental Figures 1 and 2 available online at https://doi.org/10.1155/2017/9067049 for methods and results).

### 2.2. Adipocyte Generation and Differentiation

The immortalized white preadipocyte cell line was generated using preadipocytes from omental adipose tissue obtained from a donor patient (age = 33 years, Caucasian, male, body mass index [BMI] = 44.5) without a history of smoking or diabetes (Zenbio). The immortalized brown preadipocyte cell line was generated using preadipocytes from the epicardial adipose tissue of a 62-year-old Caucasian female donor (PromoCell). Epicardial tissue is known to exhibit brown/beige phenotype due to the presence of brown adipocytes, as indicated by higher expression levels of UCP-1 in the epicardia when compared to other fat depots [20, 21].

**Figure 1:**
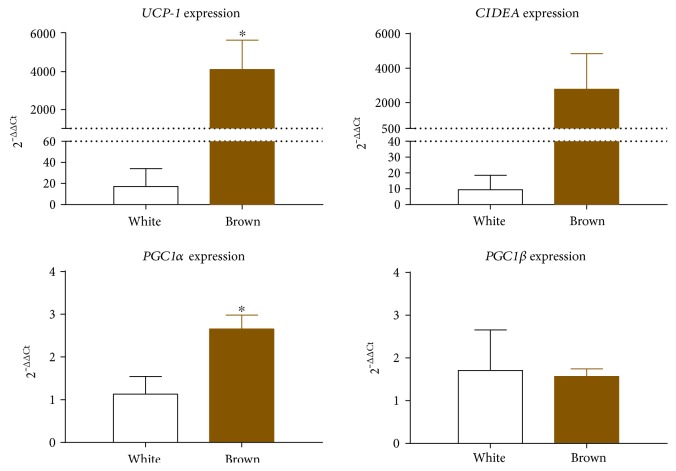
Characterization of white and brown adipocytes. Preadipocytes were cultured for 24 hours prior to differentiation for 10 days as described in Section 2. After 10 days, gene expression of *UCP-1*, *CIDEA*, *PGC1α*, and *PGC1β* was assessed by qRT-PCR from 2 to 3 biological replicates of adipocytes cultured in separate wells. Bar graphs represent mean + SEM of *N* = 2-3 biological replicates of the same adipocyte clone. Student's *t*-test, ^∗^*p* < 0.05.

Preadipocytes were immortalized by overexpressing E6 and E7 oncoproteins from the human papillomavirus as described previously [22] by infection using pLenti-III-HPV-16 E6/E7 (Applied Biological Materials, Canada), which leads to the overexpression of oncoproteins E6 and E7, and infected immortalized cells were selected using the antibiotic puromycin. Immortalized preadipocyte clones were isolated and characterized by assessing their ability to differentiate and accumulate triglyceride. Triglyceride accumulation was assessed by visualization of lipid droplets and quantitation of basal and stimulated lipolytic activity (induced by isoproterenol) (Supplemental Figure 3). In addition, expression of adipogenic genes and adipocyte markers (*CEBPα*, *PPARγ*, *FABP4*, *FASN*, *leptin*, and *ADIPQ*) was assessed (Supplemental Figure 4). In the studies described in this report, two clones for each characteristic adipocyte type (i.e., white versus brown) were used (see also additional genes in Figure 1 and Supplemental Material).

Immortalized human preadipocytes were seeded for differentiation on transwells for 6-well plates (4.67 cm^2^) at 350,000 cells/transwell, which corresponds to 75,000 cells/cm^2^. Preadipocytes were differentiated per manufacturer's protocol (Invitrogen). Adipocyte differentiation was induced with differentiation media (RPMI-based media with an in-house differentiation cocktail containing 0.5 *μ*g/ml insulin, 400 ng/ml dexamethasone, 44 *μ*g/ml IBMX, 9 ng/ml L-thyroxine, 3 *μ*g/ml ciglitazone, 8 *μ*g/ml d-biotin, and 10% FBS) for 3 days, and cells were then maintained for an additional 4 days in maintenance media (RPMI-based media, supplemented with 10% FBS, 0.5 *μ*g/ml insulin, and 8 *μ*g/ml d-biotin). On day 7, the cells were switched to a lower concentration of FBS (3%) in maintenance media (RPMI-based media supplemented with 8 *μ*g/ml d-biotin) and maintained until coculture with macrophages on day 11.

### 2.3. Adipocyte and Macrophage Coculture

On day 1, preadipocytes were seeded on transwell plate inserts and differentiated into adipocyte monocultures as described above. On day 7, in separate 6-well plates, monocytes were seeded and differentiated into macrophage monocultures as described above. On day 10, prior to coculture, PMA-differentiated macrophages were cultured for 24 hours in PMA-free growth media containing 50 ng/ml IL-4 (R&D Systems) or 5 ng/ml IFN*γ* (R&D Systems) and 10 ng/ml LPS (Sigma). On day 11, after rest (being left untreated) and stimulation (with either LPS/IFN*γ* or IL-4), macrophage monocultures were washed and the media were replenished with RPMI-based media supplemented with 3% FBS. Concomitantly, adipocyte monocultures grown on transwell inserts were washed and transferred to the six-well plates containing macrophages and then cultured for an additional 24 hours. On day 12, 24 hours after macrophage monoculture or coculturing with adipocytes, cell culture media were collected to assess cytokine secretion and macrophages were harvested for transcriptional profiling. The experimental procedure is outline in Supplemental Figure 5.

### 2.4. RNA Isolation and Gene Expression Profiling

RNA was extracted from preadipocytes and differentiated adipocytes using the Qiagen RNeasy kit (Qiagen) per manufacturer's recommendation. cDNA was synthesized with 500 ng of RNA using either the RT2 First Strand kit (Qiagen) or High-Capacity cDNA Reverse Transcription kit (Life Technologies) per manufacturer's recommendation. Quantitative PCR (qPCR) was performed using 500 ng cDNA for amplification of RPLO, as the housekeeping gene and the following genes: *UCP-1*, *CIDEA*, *PGC1α, PGC1β*, *CEBPα*, *ADIPQ*, *PPARγ*, *FABP4*, *FASN*, and leptin, using commercial primers obtained from Invitrogen. Data were analyzed using the delta delta Ct method.

RNA was extracted from macrophages using the TRIzol/chloroform method, and cDNA was synthesized using a RT2 First Strand kit (Qiagen) with 1 *μ*g. Quantitative PCR (qPCR) reactions were carried out with 500 ng cDNA using commercial primers, and data were analyzed using the delta delta Ct method with values normalized to the housekeeping gene, ribosomal 18S. The following primers were obtained from the Integrated DNA Technologies: (CCR7, Hs.PT.58.1402597), (CD36, Hs.PT.56a.3615957), (HIF1a, Hs.PT.58.534274), (IL-10, Hs.PT.58.2807216), (NFκB, Hs.PT.58.20344216), (PPAR*γ*, Hs.PT.58.25464465), (STAT3, Hs.PT.58.3750282), (STAT6, Hs.PT.58.19698584), (COX1, Hs.PT.58.47173257), and (COX2, Hs.PT.58.77266). CCL7 (Hs00171147_m1) and MCP-1 (also known as CCL2) (Sp03766212_g1) Taqman probes were obtained from Thermo Fisher Scientific.

### 2.5. IL-6 ELISA Measurements

Levels of IL-6 in cell culture supernatants were determined using the Human IL-6 Quantikine ELISA kit according to the manufacturer's instructions (R&D Systems).

### 2.6. Data Analysis and Statistics

GraphPad Prism was used for statistical analysis. For comparisons between two groups, Student's *t*-test was performed. For comparisons with 3 or more groups, one-way ANOVAs were performed, and for multifactorial comparisons, two-way ANOVAs were performed followed by post hoc analysis using Dunnett's test or Tukey's test for multiple comparisons. A *p* value of <0.05 was deemed significant.

## 3. Results

### 3.1. Brown Adipocytes Are Characterized by a Distinct Gene Expression Profile

To confirm the phenotype of adipocytes used in this study, mRNA expression of key markers that differentiates brown from white adipocytes were examined. These markers include *UCP-1*, cell death activator (*CIDEA*), and peroxisome proliferator-activated receptor gamma coactivator 1-alpha and peroxisome proliferator-activated receptor gamma coactivator 1-beta *(PGC1α* and *PGC1β*, resp.). As shown in Figure 1, brown adipocytes exhibit a significant increase in *UCP-1* and *PGC1α* gene expression compared to white adipocytes. *CIDEA* gene expression showed a trending increase in brown compared to white adipocytes (*p* = 0.08). However, expression of *PGC1β* was not significantly different between white and brown adipocytes.

To confirm that the adipocyte clones have the ability to differentiate and accumulate triglycerides, we characterized lipid droplet formation and quantified the basal and stimulated lipolytic activity (induced by isoproterenol) (Supplemental Figure 3). In the studies described in this report, we characterized an additional clone for each characteristic adipocyte type (i.e., white versus brown) to exclude a clone effect (Supplemental Figures 3 and 4 and Supplemental Tables 1 and 2). Bright field microscopy revealed an accumulation of large lipid droplets in the differentiated white adipocytes (red arrows), while in brown adipocytes, smaller cytoplasmic accumulation of lipid droplets was observed (Supplemental Figure 3A). Differentiated adipocyte clones also showed triglyceride accumulation and lypolytic activity (Supplemental Figure 3B and C). In addition, the expression of adipogenic genes and adipocyte markers (*CEBPα*, *PPARγ*, *FABP4*, *FASN*, *leptin*, and *ADIPQ*) was assessed to confirm differentiation of preadipocytes and differentiate between the white and brown clones (Supplemental Figure 4). Notably, each of the transcripts measured were significantly increased in differentiated adipocytes compared to preadipocytes (*p* < 0.05).

### 3.2. Brown Adipocytes Are Associated with a Lower Inflammatory Environment Compared to White Adipocytes

**Figure 2:**
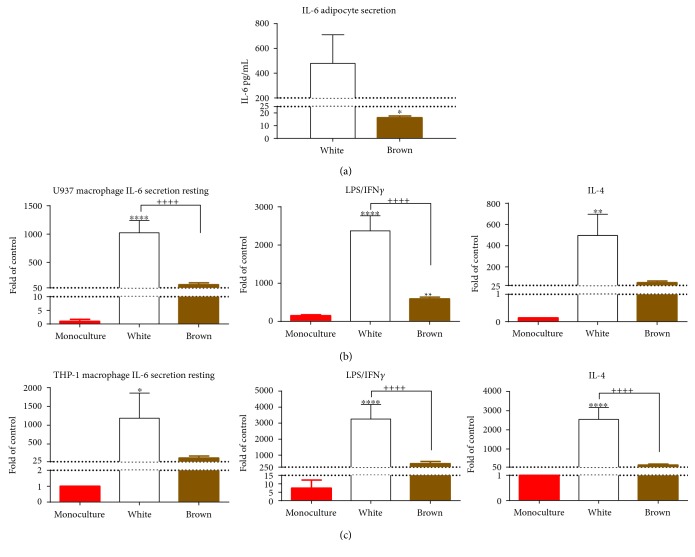
Differential effects of brown and white adipocytes on IL-6 secretion. Preadipocytes were cultured for 24 hours prior to differentiation for 10 days as described in Section 2. Differentiated adipocytes grown on transwell inserts were then cocultured for an additional 24 hours with differentiated macrophages grown on transwell plates. After 12 days, IL-6 secretion was measured from conditioned media from adipocytes. (a) White adipocyte shows significantly more IL-6 secretion than brown adipocytes. Bar graphs represent mean + SEM of *N* = 4 biological replicates of the same adipocyte clone cultured in a separate well. Student's *t*-test, ^∗^*p* < 0.05. After macrophages were differentiated, cells were either left untreated (resting state) or treated with LPS/IFN*γ* or IL-4 for 24 hours. Media with stimuli were removed and cells were then washed prior to coculture with or without adipocytes that were differentiated as described in Section 2. ((b) and (c)) Coculture of macrophages with white adipocytes significantly increases IL-6 secretion compared to monoculture of macrophage alone and when cocultured with brown adipocyte in both U937 (b) and THP-1 (c) macrophages in culture conditions of macrophages that were previously at rest or stimulated with LPS/IFN*γ* and IL-4 for 24 hours. Bar graphs represent mean + SEM of biological replicates of cultures grown in separate wells. *N* = 11 for macrophage monoculture, *N* = 4 for macrophages cocultured with white adipocytes of the same clone, and *N* = 6 for macrophages cocultured with brown adipocytes of the same clone. Two-way ANOVA followed by Tukey's post hoc test for multiple comparisons. ^∗^*p* < 0.05, ^∗∗^*p* < 0.01, and ^∗∗∗∗^*p* < 0.0001 compared to monoculture. ^++^*p* < 0.01 and ^++++^*p* < 0.0001 white versus brown coculture.

IL-6 is a cytokine that plays a major role in promoting chronic inflammation [23] and can be secreted by macrophages [24] and adipocytes [25]. Therefore, the secretory profile of IL-6 was used as a marker of inflammatory status of the adipocytes and the adipocyte and macrophage cocultures. As shown in Figure 2(a), basal secretion of IL-6 was significantly less in brown than in white adipocytes (*p* < 0.05, Figure 2(a)), suggesting that brown adipocytes have an intrinsic lower state of inflammation compared to white adipocytes. As such, the intrinsic property of the white adipocytes may confer a lower threshold of activation in response to inflammatory cues (extrinsic/environmental factors) and possibly their propensity to elicit inflammatory responses within the surrounding tissues.

The ability of macrophages to respond to environmental cues via transcriptional reprograming is well described [26]. Thus, experiments were designed to characterize the inflammatory profile of macrophages when cocultured with white and brown adipocytes. Three days postdifferentiation (i.e., resting state), U937 and THP-1 macrophage monocultures secrete very low levels of IL-6 (see Supplemental Table 1 for concentration values). However, when cocultured with adipocytes, IL-6 secretion was significantly increased when cocultured with white adipocytes (*p* < 0.05, Figure 2(b)), but not brown adipocytes. A similar pattern was observed when PMA-differentiated macrophages were subsequently stimulated with the anti-inflammatory cytokine, IL-4, and the proinflammatory molecules, LPS and IFN*γ* (Figures 2(b) and 2(c)) prior to coculture with adipocytes. In addition, when both LPS and IFN*γ* stimulated U937 and THP-1 macrophages were cocultured with brown adipocytes, there was a significant reduction in IL-1*β* secretion compared to monoculture alone (Supplemental Table 1). However, statistically significant differences in IL-1*β* secretion were not observed between macrophages cocultured with white adipocytes compared to macrophage monocultures at resting state and after IL-4 stimulation (Supplemental Table 1). These results indicate that brown adipocytes provide a lower inflammatory environment compared to that of white adipocytes.

### 3.3. Coculture with Brown Adipocytes and White Adipocytes Induces Differential Expression of Key Inflammatory Genes in Macrophages

**Figure 3:**
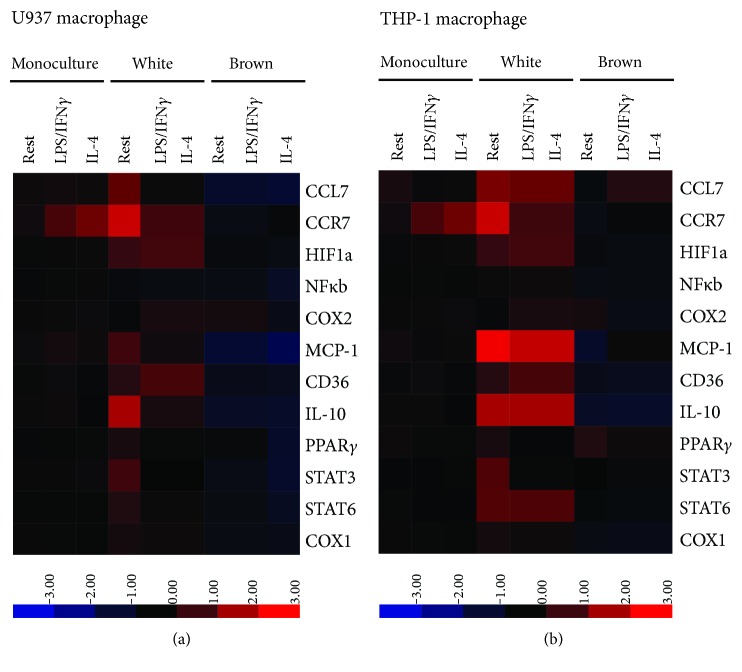
Differential effects of brown and white adipocytes on transcriptional activity. PMA-differentiated macrophages were rested (left untreated) or stimulated with LPS/IFN*γ* or IL-4 for 24 hours. Cells were then washed and cultured with or without adipocytes for an additional 24 hours (see Section 2 for differentiation and stimulation protocol). qRT-PCR analysis was used to measure expression of a panel of transcripts, which were selected for their role in macrophage inflammatory responses (see Section 4 for details). Gene expression of macrophages in adipocyte coculture was normalized to macrophages in monoculture, and the above heat maps illustrate that indirect coculture of U937 and THP-1 macrophages with white adipocytes induces a general increase in transcript expression in U937 (a) and THP-1 macrophages (b). In contrast, coculture with brown adipocytes induces a general reduction in transcript expression. Data represent biological replicates of *N* = 11 for macrophage monocultures, *N* = 4 for macrophages cocultured with white adipocytes of the same clone, and *N* = 6 for macrophages cocultured with brown adipocytes of the same clone.

To determine if the differential in secretion of cytokines (IL-6 and IL-1*β*) that is observed between macrophages that are cocultured with white compared to those with brown adipocytes could be attributed to transcriptional reprogramming, we characterized the transcriptional profile of macrophages, using a panel of inflammatory genes. Heat maps (Figure 3) illustrate the transcriptional profiles in the macrophages in response to coculture with white or brown adipocytes. Expression for each gene described in the heat map was normalized to the corresponding expression in monoculture that was exposed to the identical stimuli (i.e., at rest, LPS/IFN*γ*, or IL-4). Interestingly, coculture of macrophages with white adipocytes induced a general increase in expression of genes from the panel (denoted by a greater degree in shades of red). In contrast, when macrophages were cocultured with brown adipocytes, a general reduction of expression was observed (denoted by an increase in shades of blue). Once again, these data suggest that white adipocytes induce changes in transcriptional profiles in resting and previously stimulated macrophages, while brown adipocytes appear to dampen or even reduce expression of several key genes.

**Figure 4:**
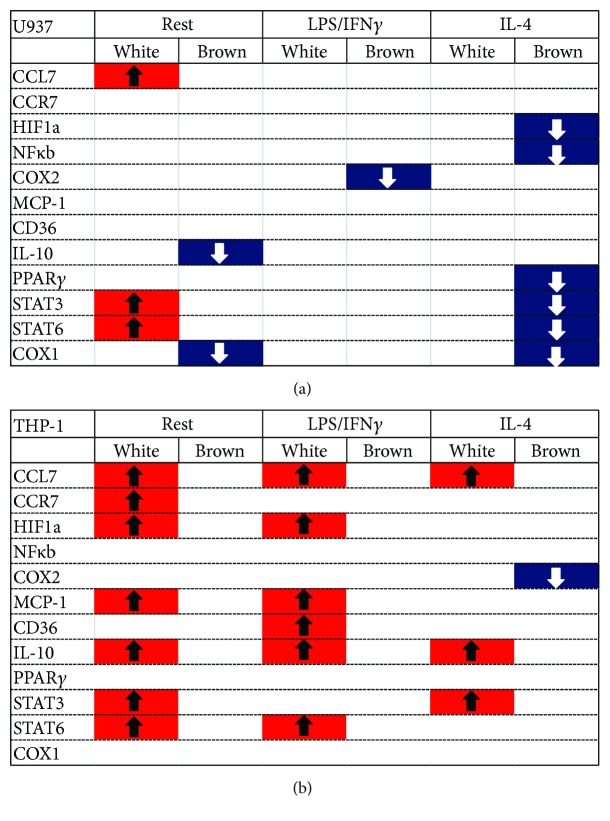
Summary of statistical analysis of transcript expression in macrophages. Summary of data analysis from Figure 3, which highlights transcripts that show statistically significant upregulation (denoted in red with up arrows) and transcripts that are significantly downregulated (denoted in blue with down arrows) in U937 macrophages (a) and THP-1 macrophages (b) when compared to monocultures of a similar stimulation state. Data were analyzed by two-way ANOVA followed by Tukey's post hoc for multiple comparisons.

Further analysis revealed that white adipocytes generally induced statistically significant changes in expression of several transcripts in both U937 and THP-1 macrophages that were previously at rest and those that were previously stimulated with LPS/IFN*γ* when compared to monocultured macrophages (*p* < 0.05, Figures 4(a) and 4(b), Supplemental Figures 6 and 7 and Supplemental Table 2). THP-1 macrophages appeared to be more sensitive to white adipocytes than U937 macrophages, as expression of several gene transcripts were altered by coculture with white adipocytes at rest and when previously treated with LPS/IFN*γ* or IL-4 (Figure 4(b)). Although U937 macrophages demonstrated changes in expression of most transcripts in the presence of white adipocytes when macrophages were previously at rest, significant changes were not observed for macrophages cocultured with white adipocytes when macrophages were previously stimulated with LPS/IFN*γ* or IL-4 (Figure 4(a)).

It is notable that in stark contrast to coculture of macrophages with white adipocytes, coculture with brown adipocytes has minimal effects on the expression of proinflammatory genes examined in the U937 macrophages. The most prominent changes in gene expression in U937 cells were observed when macrophages were cocultured with brown adipocytes after their previous exposure to IL-4. This culture condition led to a decrease in transcript levels which suggests a potential lowering in the inflammatory status of the macrophages (Figure 4(a)). In THP-1 macrophages, with the exception of COX2, coculture with brown adipocytes had no influence on the expression of inflammatory gene transcripts in THP-1 macrophages when macrophages were at rest or previously stimulated, suggesting that there are differential sensitivities among macrophages in their ability to respond to anti-inflammatory cues produced by brown adipocytes. Nevertheless, together, these data indicate differential regulation of expression of several transcripts in macrophages that are induced by the presence of white adipocytes compared to brown adipocytes. Moreover, these data demonstrate that environmental cues, which are regulated by adipocytes, dictate macrophage reprogramming of transcripts involved in inflammatory responses, with white adipocytes promoting a general upregulation and brown adipocytes dampening and/or inhibiting transcript expression.

## 4. Discussion

The function of different adipose tissue depots and their influence on metabolism is driven by anatomical and intrinsic differences between the adipocytes within the different depots [10, 11]. These intrinsic differences are the result of adipocyte developmental origin, gene expression patterns, secretory capacity, and lipolytic activity (for review, see [27]). While traditionally AT is subdivided into WAT and BAT, there is evidence that within WAT depots, there is heterogeneity. Indeed, depots that surround the internal organs are composed of both white and brown adipocytes, which have metabolically distinct phenotypes resulting in opposing biological functions [11]. For example, lypolytic activity in WAT induces the release of free fatty acid (FFA), while in BAT, FFAs are burned [28, 29]. Interestingly, an increase in FFA can induce acute inflammation and is associated with insulin resistance in humans ([30] and reviewed in [31]). In addition, BAT may be involved in protecting critical organs by providing heat through thermogenesis, which in turn, would potentially have a positive influence on metabolism (for review, see [32]). WAT can undergo browning [33], a process that is correlated with an increase in thermogenesis and can be induced by numerous factors. However, whether white adipocytes have metabolic flexibility and can transdifferentiate or if the appearance of BAT in WAT is the result of de novo adipogenesis is still under debate (for review, see [34]). Nonetheless, little is known about the relationship between brown adipocytes and inflammation and whether there are distinct and differential susceptibilities of white and brown adipocytes in promoting an inflammatory milieu, outside of the tissue microenvironment.

In the present study, white and brown adipocytes were removed from their tissue microenvironment to determine if they would retain their intrinsic properties and proclivity to influence inflammation in macrophages. Characterization of the immortalized white and brown adipocyte clones demonstrated a distinct gene expression profile (Figure 1 and Supplemental Figures 3 and 4). Consistent with prior research using the same immortalization approach [22], we show that the preadipocytes have retained both their ability to differentiate into adipocytes as well as retain their distinct properties as white and brown adipocytes, as indicated in our characterization (Figure 1 and Supplemental Figures 3 and 4). As expected, compared to white adipocytes, brown adipocytes showed a significant upregulation of *UCP-1* and *PCG1α*, while *CIDEA* showed a trending increase. *CIDEA* controls lipid droplet fusion and lipid storage in both brown and white adipocytes but is enriched in brown adipocytes [35]. *PGC1α* is involved in brown adipocyte differentiation and is a major regulator of brown adipocyte function. Lastly, *PGC1β* is primarily enriched in brown adipocytes but not in white adipocytes [36] and expressed in tissues with high-oxidative capacity. While previous studies [36] report expression in only brown adipocytes, recent data indicate that PGC1*β* may also play a major functional role in white adipocytes [37].

Circulating proinflammatory cytokines are increased in obesity, and chronic, low-grade inflammation has been shown to lead to insulin resistance and diabetes [5]. In the present study, levels of secreted IL-6 were used to determine if brown and white adipocytes would vary in their basal inflammatory profile (Figure 2). Notably, brown adipocytes secreted markedly lower levels of IL-6 compared to white adipocytes (Figure 2(a)), which indicates that brown adipocytes may provide a lower proinflammatory milieu compared to white adipocytes. While we used IL-6 as a marker of inflammatory status, it has been proposed that IL-6 secreted by or expressed in BAT may play a noninflammatory metabolic role in improving insulin responsiveness and glucose homeostasis. The differential role of IL-6 described in BAT may be mediated by activation of distinct downstream signaling pathways [38]. Thus, it is possible that IL-6 may play differential roles in BAT and WAT due to variations in expression levels of receptors and other downstream signaling proteins. In the present study, we examined IL-6 levels secreted by adipocytes in an isolated setting. Our data demonstrate quantitative differences (~80-fold) between white and brown adipocytes in secreted IL-6 levels, and these differences correlate to altered responses of macrophages. However, more work is needed to identify additional factors that may be contributing to the macrophage response to white versus brown adipocytes in this setting.

It is important to note that the present study is not without limitations. First, it is possible that our observations could be mediated by a clone effect. To exclude this possibility, we generated and examined an additional white and brown adipocyte clone. Additional data from these clones demonstrated a similar inflammatory profile in IL-6 secretion and macrophage response without significant differences between the clones (Supplemental Tables 1 and 2). These results provide support for an intrinsic difference between the white and brown adipocytes, although additional clones from different patients should be tested to definitively conclude this. Second, we cannot exclude a possible obesity effect as the white adipocyte clone was obtained from an obese donor while the BMI of the donor for the brown adipocyte clone is unknown. However, while adipose tissue inflammation is associated with insulin resistance, it has been reported that AT inflammation appears to be independent of BMI [39, 40]. Lastly, it is possible that the observed differences between the white and brown adipocyte clones could be mediated by gender. The white adipocytes were derived from a male donor while the brown adipocytes were derived from a female donor. Indeed, sexual dimorphism in distribution and function of AT depots has been described, in particular for WAT [41]. Based on animal studies, the effect of sex on AT characteristics affected by a high-fat diet, including their inflammatory profile, appears to be modulated at the hormonal level [42]. However, it is possible that we may have limited the potential confound of sex hormones given that our studies were conducted on isolated adipocytes outside of their tissue microenvironment.

Macrophages contribute to adipose tissue homeostasis, and the influence of adipose tissue macrophages (ATMs) on adipocyte function is increasingly becoming appreciated (for recent review, see [43]). ATMs are higher in the depots that surround the internal organs than subcutaneous fat, and an increase in ATMs occurs with increased body weight, and this increase in macrophage burden correlates with insulin resistance [44]. In the present study, the indirect coculture of macrophages with adipocytes revealed differential influence of white and brown adipocytes on secreted IL-6 levels of the coculture. Indeed, this was supported by additional data, in which macrophages previously stimulated with LPS and IFN*γ* and subsequently cocultured with brown adipocytes resulted in a significant reduction in IL-1*β* secretion compared to macrophages in monoculture (Supplemental Table 1). The inability to detect differences in IL-1*β* secretion between white and brown adipocytes cocultured macrophages at resting state, and after stimulation with IL-4 suggests that macrophage-mediated IL-1*β* secretion is possibly responding to factor(s) released from brown adipocytes that are induced by LPS and IFN*γ* treatment. This is likely the case given that IL-1*β* levels from macrophages cocultured with white adipocytes were not significantly different from monocultured macrophages under any condition examined. IL-1*β* detected in conditioned media is macrophage derived as both types of adipocytes did not secrete IL-1*β*. These data implicate that white and brown adipocytes provide different stimulatory environments for the macrophages. Furthermore, the results also demonstrate that white and brown adipocytes retain their intrinsic pro- and anti-inflammatory influence outside of their tissue microenvironment.

The varied levels of IL-6 secretion and thus the potential difference in inflammatory environment between white and brown adipocytes imply that they would therefore differ in their influence on the macrophages themselves, for example, via macrophage transcriptional reprograming [45]. Prior studies have shown that macrophages isolated from murine BAT following adoptive transfer experiments have reduced expression of cytokine, chemokine, and receptor transcripts compared to their in vitro counterparts or those isolated from WAT [17]. Similarly, in the present study, white adipocytes induced expression of several inflammatory transcripts in macrophages, while brown adipocytes either reduced or did not significantly affect gene expression in both U937 and THP-1 macrophages regardless of stimulation state (Figures 3 and 4). Our results point to culture conditions as a strong driver of transcriptional regulation of macrophages. In addition, the lower impact of stimulus (i.e., LPS/IFN*γ* and IL-4) on transcriptional changes in macrophages could be attributed to our study design. In the present studies, macrophages were exposed to stimuli for 24 hours and washed to remove stimuli prior to coculturing with adipocytes. Thus, the limited effect of LPS/IFN*γ* or IL-4 is possibly due to the removal of stimulus. Although many of the transcripts have been described as either proinflammatory (NFκB, COX2, and MCP-1) or anti-inflammatory markers (e.g., IL-10, PPAR*γ*), the traditional dichotomous role on inflammation for some of these markers has been questioned (e.g., NFκb) [46]. Moreover, recent work has highlighted that the inflammatory status and polarization of macrophages appear to lie along a spectrum that is defined by the complex expression of a number of markers [26, 47]. Thus, based on the limited number of classically defined pro- versus anti-inflammatory markers, we caution drawing conclusions that an increase in markers such as MCP-1 or COX2 is suggestive of a proinflammatory state or that an increase in PPAR*γ* is indicative of an anti-inflammatory state, as there may be a complex interplay among the markers to promote a proinflammatory or anti-inflammatory state. Nonetheless, taken together, these results suggest that white adipocytes have the potential to alter inflammatory status in macrophages, as indicated by marked changes in their transcriptional profile, while brown adipocytes have little to no impact.

A few key observations can be made from further analysis of the differential profile of transcripts expressed in macrophages, that is, those that are significantly affected by white and brown adipocyte cocultures (Figure 4 and Supplemental Figures 6 and 7). First, compared to brown adipocytes, white adipocytes induce upregulation of CCL7, STAT3, and STAT6 in both resting U937 and THP-1 macrophages. Adipose tissue inflammation results in recruitment of monocytes due to an increase in chemokine expression [48]. The chemoattractant proteins MCP-1 and CCL7/MCP-3 have been shown to be upregulated in the adipose tissue of obese subjects and ATMs isolated from obese mice [49]. The STAT signaling axis has been shown to be protective and required for alternative activation of macrophages in adaptive thermogenesis [50], and activation of the transcription factors STAT3 and 6 could be in response to the proinflammatory environment provided by the white adipocytes. For example, leptin is released from WAT, and leptin has been shown to activate STAT isoforms and, in particular, STAT3 in macrophages [51]. Second, in the IL-4-stimulated state, white adipocytes do not have a strong effect on U937 macrophages as brown adipocytes do. This could be the result of the complex interplay of intrinsic differentiation factors and environmental input, which determines macrophage fate [52]. Third, coculture with brown adipocytes does not significantly affect transcriptional regulation in THP-1 macrophages across all stimulation states but generally does for resting and IL-4-stimulated U937 macrophages. This could be the result of differences between the two monocyte cell lines. Indeed, we observed that for a number of transcripts, THP-1 macrophages have slightly but statistically significant lower levels of expression, based upon higher Ct values (data not shown). Alternatively, THP-1 macrophages may be less responsive to the environmental cues provided by brown than white adipocytes.

Previous studies of WAT browning suggest that improvement in metabolic function is due to thermogenesis [53]. However, the results here suggest that brown adipocytes, by providing a less inflammatory environment, may be inducing a quiescent macrophage state, which, if extrapolated to the in vivo setting, would result in a less inflammatory macrophage profile and lead to decreased inflammation in AT. It is possible that thermogenic properties of brown adipocytes are not exclusive from its ability to provide a reduced inflammatory environment. Prior studies demonstrated that increased circulating FFA is associated with inflammation [30], as such the propensity for brown adipocytes to metabolize FFA as a fuel source for thermogenesis [29, 54] suggests that reduced FFA from brown adipocytes may be contributing to their lower inflammatory profile compared to that of white adipocytes. Notably, the ability of brown adipocytes to dampen inflammatory responses in macrophages suggests that brown adipocytes may provide some protection against inflammation. Moreover, these data suggest that factors secreted from brown adipocytes or from browning WAT may have potential therapeutic efficacy against a spectrum of inflammatory conditions including adipose tissue inflammation.

Our observations imply that a model based on the differential effects of white and brown adipocytes on macrophages represents a unique opportunity to delineate novel mechanisms, targets, and secreted biomolecules that regulate cellular and pathological inflammation. In this regard, employing a platform, which entails multiomics (functional proteomic-activity enrichment, phosphoproteomics, and lipidomics) and Bayesian causal network methodologies [55], represents an approach to understand the role of BAT and WAT in conferring inflammatory states. This type of study may lead to a greater understanding of the inflammatory mechanisms and regulation of the pathways associated with obesity.

## Supplementary Material

Supplemental Figure 1. Characterization of surface markers for differentiated macrophages. Supplemental Figure 2. Characterization of phagocytosis in differentiated macrophages. Supplemental Figure 3. Characterization of adipocyte differentiation. Supplemental Figure 4. Characterization of gene expression in differentiated adipocyte clones. Supplemental Figure 5. Schematic of Experimental Procedure. Supplemental Figure 6. Quantitative analysis of transcript expression of inflammatory genes in U937 macrophages. Supplemental Figure 7. Quantitative analysis of transcript expression of inflammatory genes in THP-1 macrophages. Supplemental Table 1. Inflammatory cytokine secretion in two different clones of white and brown adipocytes. Supplemental Table 2. Expression of Inflammatory genes in macrophages after co-culture with two different clones of white and brown adipocytes.

## Abbreviations

AT: Adipose tissue
BAT: Brown adipose tissue
DIO: Diet-induced obesity
LPS: Lipopolysaccharide
Interferon-*γ*: IFN*γ*
IL-4: Interleukin-4
IL-6: Interleukin-6
PMA: Phorbol 12-myristate 13-acetate
WAT: White adipose tissue.
