# Supplementary material for: Intrinsic Properties of Brown and White Adipocytes Have Differential Effects on Macrophage Inflammatory Responses
Source: Mediators Inflamm. 2017 Mar 26;2017:9067049. doi: 10.1155/2017/9067049 (PMC5385256; doi:10.1155/2017/9067049)
Supplement: Supplementary file 5 [file 9067049.f5.pptx]

## Slide 1
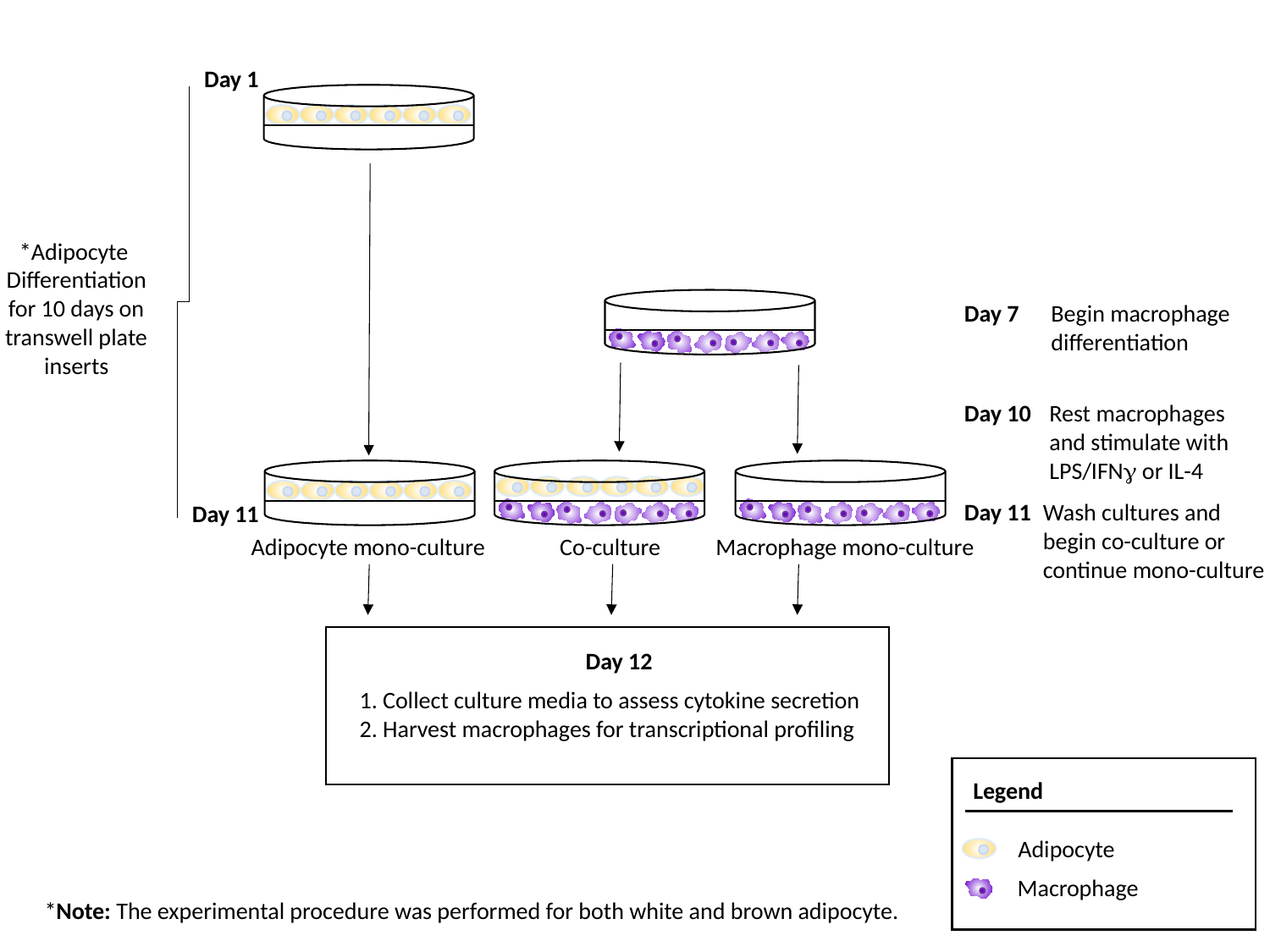

Day 1
*Adipocyte
Differentiation for 10 days on transwell plate inserts
Day 7
Begin macrophage
differentiation
Day 10
Rest macrophages and stimulate with LPS/IFNg or IL-4
Wash cultures and begin co-culture or continue mono-culture
Day 11
Day 11
Adipocyte mono-culture
Co-culture
Macrophage mono-culture
Day 12
1. Collect culture media to assess cytokine secretion
2. Harvest macrophages for transcriptional profiling
Legend
Adipocyte
Macrophage
*Note: The experimental procedure was performed for both white and brown adipocyte.
