# Supplementary material for: Intrinsic Properties of Brown and White Adipocytes Have Differential Effects on Macrophage Inflammatory Responses
Source: Mediators Inflamm. 2017 Mar 26;2017:9067049. doi: 10.1155/2017/9067049 (PMC5385256; doi:10.1155/2017/9067049)
Supplement: Supplementary file 6 [file 9067049.f6.docx]

**Supplemental Table 1. Inflammatory cytokine secretion in two different clones of white and brown adipocytes**

|  | | **White** | | **p-value** | **Brown** | | **p-value** | **U937 mono-culture** | **THP1 mono-culture** |
| --- | --- | --- | --- | --- | --- | --- | --- | --- | --- |
|  |  | **Clone 1** | **Clone 2** |  | **Clone 1** | **Clone 2** |  |  |  |
| **IL-6 secretion Adipocyte Monoculture** | | 479 ± 230.9 pg/mL |  |  | 16.25 ± 1.377 pg/mL |  |  | NA | NA |
| **IL-6 secretion** | | | | | | | | | |
| U937 co-culture | Rest | 5151 ± 1094 pg/mL | 6595 ± 887 pg/mL | p = 0.35 | 529.7 ± 173.2 pg/mL | 590.5 ± 130.9 pg/mL | p = 0.79 | 7.5 ± 3.863 pg/mL | NA |
|  | LPS/IFNƴ | 11922 ± 1996 pg/mL | 10563 ± 917 pg/mL | p = 0.56 | 2969 ± 235.5 pg/mL | 3521 ± 237.6 pg/mL | p = 0.13 | 864.3 ± 119.6 pg/mL | NA |
|  | IL-4 | 2494 ± 1013 pg/mL | 1865 ± 200.5 pg/mL | p = 0.56 | 261.4 ± 77.06 pg/mL | 595.5 ± 116.2 pg/mL | p = 0.03^*^ | 0.7 ± 0 pg/mL | NA |
| THP-1 co-culture | Rest | 829.4 ± 470.8 pg/mL | 981.9 ± 185 pg/mL | p = 0.77 | 88.44 ± 35.85 pg/mL | 37.23 ± 14.58 pg/mL | p = 0.22 | NA | 0.7 ± 0 pg/mL |
|  | LPS/IFNƴ | 2279 ± 639.7 pg/mL | 2680 ± 313.1 pg/mL | p = 0.59 | 329.7 ± 93.84 pg/mL | 535.1 ± 161.4 pg/mL | p = 0.30 | NA | 7.789 ± 3.472 pg/mL |
|  | IL-4 | 1782 ± 433.8 pg/mL | 1434 ± 162.2 pg/mL |  | 114.5 ± 35.58 pg/mL | 126.6 ± 37.08 pg/mL |  | NA | 0.7 ± 0 pg/mL |
| **IL-1β secretion^**^** | | | | | | | | | |
| U937 co-culture | Rest | 23.07 ± 2.894 pg/mL | 24.57 ± 5.02 pg/mL | p = 0.80 | 9.462 ± 1.969 pg/mL | 9.772 ± 1.969 pg/mL | p = 0.93 | 23.88 ± 3.013 pg/mL | NA |
|  | LPS/IFNƴ | 524.8 ± 85.15 pg/mL | 548.4 ± 75.31 pg/mL | p = 0.84 | 189.6 ± 28.25 pg/mL | 226.9 ± 17.49 pg/mL | p = 0.29 | 508.1 ± 62.36 pg/mL | NA |
|  | IL-4 | 8.448 ± 2.903 pg/mL | 6.743 ± 1.613 pg/mL | p = 0.63 | 8.843 ± 2.193 pg/mL | 8.125 ± 1.559 pg/mL | p = 0.80 | 9.063 ± 1.599 pg/mL | NA |
| THP-1 co-culture | Rest | 0.878 ± 0.123 pg/mL | 1.665 ± 0.895 pg/mL | p = 0.42 | 2.27 ± 1.094 pg/mL | 2.78 ± 2.154 pg/mL | p = 0.85 | NA | 1.494 ± 0.43 pg/mL |
|  | LPS/IFNƴ | 15.44 ± 4.847 pg/mL | 23.14 ± 2.312 pg/mL | p = 0.20 | 11.71 ± 2.094 pg/mL | 8.973 ± 2.293 pg/mL | p = 0.40 | NA | 15.31 ± 2.245 pg/mL |
|  | IL-4 | 1.323 ± 0.323 pg/mL | 0.763 ± 0.237 pg/mL | p = 0.25 | 5.315 ± 1.633 pg/mL | 5.255 ± 3.206 pg/mL | p = 0.99 | NA | 1.614 ± 0.253 pg/mL |

Data are expressed as Mean ± SEM in pg/mL. Student’s t test was used to compare the two white or brown adipocyte clones. In general, there were no significant differences between clones. * p < 0.05. ** IL-1β was measured using the human IL-1β/IL-1F2 Quantikine ELISA kit (R&D systems) per manufacturer’s recommendation. The sensitivity of IL-1β ELISA assay is 1 pg/mL.
