# Supplementary material for: Intrinsic Properties of Brown and White Adipocytes Have Differential Effects on Macrophage Inflammatory Responses
Source: Mediators Inflamm. 2017 Mar 26;2017:9067049. doi: 10.1155/2017/9067049 (PMC5385256; doi:10.1155/2017/9067049)
Supplement: Supplementary file 7 [file 9067049.f7.docx]

**Supplemental Table 2. Expression of Inflammatory genes in macrophages after co-culture with two different clones of white and brown adipocytes**

|  | | White | | p-value | Brown | | p-value | U937 mono-culture | THP-1 mono-culture |
| --- | --- | --- | --- | --- | --- | --- | --- | --- | --- |
|  |  | Clone 1 | Clone 2 |  | Clone 1 | Clone 2 |  |  |  |
| **CCL7** | | | | | | | | | |
| U937 co-culture | Rest | 2.52 ± 1.13 | 2.21 ± 0.80 | p = 0.83 | 0.38 ± 0.11 | 0.47 ± 0.17 | p = 0.66 | 1.22 ± 0.24 | NA |
|  | LPS/IFNƴ | 1.14 ± 0.60 | 1.17 ± 0.54 | p = 0.97 | 0.25 ± 0.04 | 0.19 ± 0.05 | p = 0.34 | 1.31 ± 0.27 | NA |
|  | IL-4 | 1.25 ± 0.44 | 0.91 ± 0.13 | p = 0.49 | 0.35 ± 0.09 | 0.30 ± 0.07 | p = 0.64 | 1.22 ± 0.21 | NA |
| THP-1 co-culture | Rest | 3.00 ± 0.59 | 4.41 ± 2.14 | p = 0.55 | 0.81 ± 0.20 | 1.03 ± 0.27 | p = 0.51 | NA | 1.47 ± 0.47 |
|  | LPS/IFNƴ | 2.68 ± 0.64 | 3.01 ± 1.15 | p = 0.81 | 1.68 ± 0.50 | 1.51 ± 0.40 | p = 0.80 | NA | 1.12 ± 0.15 |
|  | IL-4 | 2.88 ± 0.54 | 1.38 ± 0.10 | p = 0.03* | 0.67 ± 0.18 | 0.72 ± 0.28 | p = 0.89 | NA | 1.14 ± 0.14 |
| **CCR7** | | | | | | | | | |
| U937 co-culture | Rest | 0.30 ± 0.10 | 2.21 ± 0.36 | p = 0.13 | 0.40 ± 0.11 | 0.90 ± 0.27 | p = 0.12 | 1.36 ± 0.30 | NA |
|  | LPS/IFNƴ | 1.28 ± 0.56 | 0.67 ± 0.19 | p = 0.34 | 0.61 ± 0.10 | 0.95 ± 0.33 | p = 0.35 | 1.43 ± 0.33 | NA |
|  | IL-4 | 1.20 ± 0.36 | 3.36 ± 2.24 | p = 0.38 | 0.32 ± 0.15 | 0.40 ± 0.05 | p = 0.63 | 1.27 ± 0.23 | NA |
| THP-1 co-culture | Rest | 4.98 ± 2.01 | 1.03 ± 0.20 | p = 0.10 | 0.70 ± 0.36 | 0.47 ± 0.28 | p = 0.62 | NA | 1.26 ±0.24 |
|  | LPS/IFNƴ | 2.05 ± 0.80 | 1.27 ± 0.64 | p = 0.48 | 0.94 ± 0.38 | 1.17 ± 0.43 | p = 0.69 | NA | 2.14 ± 0.65 |
|  | IL-4 | 1.33 ± 0.54 | 2.19 ± 1.65 | p = 0.64 | 0.58 ± 0.27 | 0.42 ± 0.08 | p = 0.58 | NA | 2.78 ± 1.27 |
| **HIF1a** | | | | | | | | | |
| U937 co-culture | Rest | 1.59 ± 0.47 | 1.58 ± 0.29 | p = 0.99 | 0.75 ± 0.14 | 1.06 ± 0.20 | p = 0.23 | 1.11 ± 0.16 | NA |
|  | LPS/IFNƴ | 1.46 ± 0.47 | 0.87 ± 0.53 | p = 0.45 | 0.75 ± 0.13 | 0.67 ± 0.12 | p = 0.66 | 1.12 ± 0.15 | NA |
|  | IL-4 | 1.05 ± 0.22 | 0.66 ± 0.11 | p = 0.17 | 0.34 ± 0.08 | 0.71 ± 0.10 | p = 0.02* | 1.05 ± 0.09 | NA |
| THP-1 co-culture | Rest | 1.91 ± 0.09 | 1.34 ± 0.37 | p = 0.19 | 0.84 ± 0.06 | 0.84 ± 0.13 | p = 0.99 | NA | 1.08 ± 0.12 |
|  | LPS/IFNƴ | 2.07 ± 0.47 | 2.43 ± 0.77 | p = 0.71 | 0.71 ± 0.03 | 0.70 ± 0.13 | p = 0.91 | NA | 1.09 ± 0.12 |
|  | IL-4 | 1.09 ± 0.27 | 1.14 ± 0.35 | p = 0.91 | 0.79 ± 0.15 | 0.71 ± 0.08 | p = 0.66 | NA | 1.14 ± 0.19 |
| **NFkB** | | | | | | | | | |
| U937 co-culture | Rest | 0.83 ± 0.13 | 1.28 ± 0.31 | p = 0.22 | 0.64 ± 0.12 | 0.82 ± 0.12 | p = 0.33 | 1.06 ± 0.09 | NA |
|  | LPS/IFNƴ | 0.74 ± 0.22 | 0.92 ± 0.23 | p = 0.60 | 1.09 ± 0.23 | 1.12 ± 0.20 | p = 0.92 | 1.08 ± 0.12 | NA |
|  | IL-4 | 0.98 ± 0.25 | 0.76 ± 0.25 | p = 0.56 | 0.42 ± 0.06 | 0.51 ± 0.10 | p = 0.42 | 1.09 ± 0.13 | NA |
| THP-1 co-culture | Rest | 1.13 ± 0.12 | 1.76 ± 0.19 | p = 0.03* | 0.65 ± 0.06 | 0.57 ± 0.09 | p = 0.44 | NA | 1.04 ± 0.08 |
|  | LPS/IFNƴ | 1.21 ± 0.43 | 0.81 ± 0.19 | p = 0.43 | 0.75 ± 0.05 | 0.82 ± 0.11 | p = 0.53 | NA | 1.07 ± 0.11 |
|  | IL-4 | 1.48 ± 0.31 | 1.27 ± 0.30 | p = 0.64 | 1.05 ± 0.11 | 0.88 ± 0.07 | p = 0.24 | NA | 1.04 ± 0.08 |
| **CD36** | | | | | | | | | |
| U937 co-culture | Rest | 1.25 ± 0.54 | 1.56 ± 0.36 | p = 0.65 | 1.49 ± 0.23 | 2.18 ± 0.35 | p = 0.13 | 1.09 ± 0.14 | NA |
|  | LPS/IFNƴ | 0.81 ± 0.20 | 0.81 ± 0.31 | p = 0.99 | 1.52 ± 0.20 | 1.44 ± 0.20 | p = 0.79 | 1.09 ± 0.12 | NA |
|  | IL-4 | 1.31 ± 0.32 | 0.61 ± 0.07 | p = 0.08 | 0.77 ± 0.14 | 1.49 ± 0.25 | p = 0.03* | 1.11 ± 0.14 | NA |
| THP-1 co-culture | Rest | 1.70 ± 0.21 | 1.20 ± 0.34 | p = 0.26 | 0.55 ± 0.07 | 0.54 ± 0.06 | p = 0.86 | NA | 1.08 ± 0.12 |
|  | LPS/IFNƴ | 2.12 ± 0.84 | 2.51 ± 0.82 | p = 0.75 | 0.50 ± 0.08 | 0.52 ± 0.10 | p = 0.92 | NA | 1.16 ± 0.18 |
|  | IL-4 | 1.43 ± 0.36 | 1.32 ± 0.30 | p = 0.83 | 0.56 ± 0.10 | 0.69 ± 0.06 | p = 0.33 | NA | 1.06 ± 0.11 |
| **IL-10** | | | | | | | | | |
| U937 co-culture | Rest | 1.47 ± 0.45 | 1.48 ± 0.29 | p = 0.99 | 0.31 ± 0.08 | 0.41 ± 0.07 | p = 0.38 | 1.16 ± 0.17 | NA |
|  | LPS/IFNƴ | 1.46 ± 0.59 | 1.20 ± 0.62 | p = 0.78 | 0.54 ± 0.13 | 0.56 ± 0.15 | p = 0.92 | 1.27 ± 0.26 | NA |
|  | IL-4 | 0.98 ± 0.18 | 0.62 ± 0.16 | p = 0.17 | 0.42 ± 0.08 | 0.80 ± 0.10 | p = 0.02* | 1.10 ± 0.14 | NA |
| THP-1 co-culture | Rest | 4.02 ± 1.08 | 2.32 ± 1.12 | p = 0.32 | 0.43 ± 0.05 | 0.57 ± 0.09 | p = 0.22 | NA | 1.13 ± 0.15 |
|  | LPS/IFNƴ | 3.98 ± 1.90 | 5.35 ± 2.65 | p = 0.69 | 0.40 ± 0.08 | 0.30 ± 0.08 | p = 0.40 | NA | 1.13 ± 0.15 |
|  | IL-4 | 4.68 ± 1.12 | 3.16 ± 0.50 | p = 0.29 | 0.43 ± 0.09 | 0.48 ± 0.07 | p = 0.61 | NA | 1.03 ± 0.06 |
| **PPARƴ** | | | | | | | | | |
| U937 co-culture | Rest | 1.47 ± 0.41 | 1.56 ± 0.38 | p = 0.88 | 0.87 ± 0.11 | 1.33 ± 0.24 | p = 0.11 | 1.06 ± 0.12 | NA |
|  | LPS/IFNƴ | 1.07 ± 0.20 | 1.09 ± 0.27 | p = 0.94 | 1.01 ± 0.16 | 1.73 ± 0.79 | p = 0.39 | 1.04 ± 0.09 | NA |
|  | IL-4 | 0.78 ± 0.17 | 0.43 ± 0.07 | p = 0.12 | 0.39 ± 0.08 | 0.56 ± 0.18 | p = 0.40 | 1.08 ± 0.13 | NA |
| THP-1 co-culture | Rest | 1.47 ± 0.40 | 0.93 ± 0.30 | p = 0.32 | 1.62 ± 0.30 | 1.33 ± 0.21 | p = 0.46 | NA | 1.21 ± 0.22 |
|  | LPS/IFNƴ | 1.03 ± 0.28 | 1.96 ± 0.45 | p = 0.13 | 1.22 ± 0.09 | 1.30 ± 0.36 | p = 0.83 | NA | 1.08 ± 0.11 |
|  | IL-4 | 0.92 ± 0.18 | 1.15 ± 0.28 | p = 0.51 | 1.37 ± 0.35 | 1.17 ± 0.18 | p = 0.62 | NA | 1.07 ± 0.12 |
| **Stat3** | | | | | | | | | |
| U937 co-culture | Rest | 2.06 ± 0.54 | 1.51 ± 0.31 | p = 0.42 | 0.63 ± 0.09 | 0.79 ± 0.13 | p = 0.34 | 1.10 ± 0.13 | NA |
|  | LPS/IFNƴ | 1.01 ± 0.29 | 1.18 ± 0.29 | p = 0.69 | 0.68 ± 0.09 | 0.78 ± 0.11 | p = 0.52 | 1.09 ± 0.12 | NA |
|  | IL-4 | 1.20 ± 0.38 | 0.72 ± 0.09 | p = 0.27 | 0.38 ± 0.07 | 0.50 ± 0.07 | p = 0.24 | 1.12 ± 0.16 | NA |
| THP-1 co-culture | Rest | 2.26 ± 0.43 | 1.96 ± 0.56 | p = 0.68 | 1.00 ± 0.08 | 0.82 ± 0.13 | p = 0.26 | NA | 1.03 ± 0.07 |
|  | LPS/IFNƴ | 1.05 ± 0.18 | 1.23 ± 0.17 | p = 0.49 | 0.88 ± 0.09 | 1.02 ± 0.12 | p = 0.38 | NA | 1.03 ± 0.06 |
|  | IL-4 | 3.66 ± 0.76 | 1.89 ± 0.41 | p = 0.08 | 1.29 ± 0.22 | 1.02 ± 0.07 | p = 0.29 | NA | 1.04 ± 0.09 |
| **Stat6** | | | | | | | | | |
| U937 co-culture | Rest | 1.62 ± 0.38 | 1.18 ± 0.29 | p = 0.40 | 0.75 ± 0.09 | 0.95 ± 0.12 | p = 0.22 | 1.04 ± 0.08 | NA |
|  | LPS/IFNƴ | 1.15 ± 0.40 | 1.04 ± 0.34 | p = 0.85 | 0.67 ± 0.08 | 0.78 ± 0.14 | p = 0.49 | 1.05 ± 0.10 | NA |
|  | IL-4 | 1.04 ± 0.17 | 0.75 ± 0.10 | p = 0.19 | 0.46 ± 0.06 | 0.74 ± 0.12 | p = 0.06 | 1.04 ± 0.08 | NA |
| THP-1 co-culture | Rest | 2.30 ± 0.40 | 1.34 ± 0.38 | p = 0.13 | 0.88 ± 0.08 | 0.72 ± 0.08 | p = 0.18 | NA | 1.08 ± 0.14 |
|  | LPS/IFNƴ | 2.24 ± 0.47 | 1.74 ± 0.36 | p = 0.43 | 0.80 ± 0.07 | 1.06 ± 0.12 | p = 0.08 | NA | 1.02 ± 0.06 |
|  | IL-4 | 1.46 ± 0.18 | 1.55 ± 0.23 | p = 0.79 | 0.93 ± 0.16 | 1.08 ± 0.06 | p = 0.42 | NA | 1.02 ± 0.05 |
| **COX1** | | | | | | | | | |
| U937 co-culture | Rest | 0.86 ± 0.13 | 1.69 ± 0.48 | p = 0.15 | 0.50 ± 0.08 | 0.69 ± 0.17 | p = 0.34 | 1.10 ± 0.13 | NA |
|  | LPS/IFNƴ | 1.28 ± 0.45 | 1.66 ± 0.30 | p = 0.50 | 0.58 ± 0.13 | 0.77 ± 0.20 | p = 0.45 | 1.04 ± 0.09 | NA |
|  | IL-4 | 0.81 ± 0.20 | 0.83 ± 0.18 | p = 0.95 | 0.40 ± 0.08 | 0.56 ± 0.17 | p = 0.41 | 1.13 ± 0.14 | NA |
| THP-1 co-culture | Rest | 1.34 ± 0.33 | 1.31 ± 0.28 | p = 0.94 | 0.70 ± 0.09 | 0.62 ± 0.06 | p = 0.46 | NA | 1.09 ± 0.13 |
|  | LPS/IFNƴ | 1.20 ± 0.43 | 1.06 ± 0.20 | p = 0.77 | 0.58 ± 0.06 | 0.62 ± 0.06 | p = 0.61 | NA | 1.07 ± 0.11 |
|  | IL-4 | 1.30 ± 0.23 | 1.06 ± 0.15 | p = 0.41 | 0.75 ± 0.17 | 0.59 ± 0.06 | p = 0.41 | NA | 1.04 ± 0.09 |
| **COX2** | | | | | | | | | |
| U937 co-culture | Rest | 2.07 ± 0.77 | 2.29 ± 0.48 | p = 0.82 | 0.41 ± 0.08 | 0.58 0.14 | p = 0.33 | 1.19 ± 0.19 | NA |
|  | LPS/IFNƴ | 2.21 ± 0.99 | 2.67 ± 0.99 | p = 0.75 | 0.21 ± 0.04 | 0.19 0.03 | p = 0.57 | 1.31 ± 0.28 | NA |
|  | IL-4 | 1.48 ± 0.38 | 1.06 ± 0.31 | p = 0.43 | 0.76 ± 0.24 | 0.97 0.20 | p = 0.50 | 1.18 ± 0.18 | NA |
| THP-1 co-culture | Rest | 0.94 ± 0.11 | 1.57 ± 0.37 | p = 0.15 | 1.32 ± 0.25 | 0.87 0.22 | p = 0.21 | NA | 1.09 ± 0.12 |
|  | LPS/IFNƴ | 1.45 ± 0.63 | 1.76 ± 0.58 | p = 0.73 | 0.59 ± 0.15 | 0.59 0.11 | p = 0.97 | NA | 1.13 ± 0.16 |
|  | IL-4 | 1.51 ± 0.41 | 2.26 ± 0.81 | p = 0.44 | 2.84 ± 0.89 | 2.85 0.82 | p = 0.99 | NA | 1.17 ± 0.18 |
| **MCP-1** | | | | | | | | | |
| U937 co-culture | Rest | 2.05 ± 0.70 | 1.88 ± 0.77 | p = 0.88 | 0.35 ± 0.06 | 0.53 0.12 | p = 0.23 | 1.17 ± 0.20 | NA |
|  | LPS/IFNƴ | 1.25 ± 0.70 | 1.58 ± 0.90 | p = 0.79 | 0.55 ± 0.10 | 0.43 0.11 | p = 0.46 | 1.36 ± 0.32 | NA |
|  | IL-4 | 1.60 ± 0.53 | 0.77 ± 0.14 | p = 0.18 | 0.25 ± 0.10 | 0.43 0.13 | p = 0.27 | 1.21 ± 0.21 | NA |
| THP-1 co-culture | Rest | 6.42 ± 1.84 | 6.07 ± 2.15 | p = 0.91 | 0.39 ± 0.11 | 0.36 0.08 | p = 0.82 | NA | 1.25 ± 0.24 |
|  | LPS/IFNƴ | 4.76 ± 0.86 | 4.56 ± 1.78 | p = 0.92 | 1.09 ± 0.30 | 1.43 0.46 | p = 0.56 | NA | 1.12 ± 0.13 |
|  | IL-4 | 1.98 ± 0.42 | 1.28 ± 0.39 | p = 0.27 | 0.23 ± 0.06 | 0.34 0.11 | p = 0.38 | NA | 1.15 ± 0.20 |

Transcript expression was assessed by quantitative RT-PCR and data were normalized to the housekeeping gene, ribosomal 18S. Data are expressed as Mean ± SEM of 2^-ΔΔCt^ of n = 4-14 biological replicates. Student’s t test was used to compare the two white or brown adipocyte clones. In general, there were no significant differences between clones. * p < 0.05. NA = not applicable.
